# Phloroglucinol protects retinal pigment epithelium and photoreceptor against all‐*trans*‐retinal–induced toxicity and inhibits A2E formation

**DOI:** 10.1111/jcmm.12857

**Published:** 2016-04-12

**Authors:** David Cia, Aurélie Cubizolle, Céline Crauste, Nathalie Jacquemot, Laurent Guillou, Claire Vigor, Claire Angebault, Christian P. Hamel, Joseph Vercauteren, Philippe Brabet

**Affiliations:** ^1^ Laboratoire de Biophysique NeurosensorielleUMR INSERM 1107 Facultés de Médecine et de PharmacieClermont‐FerrandFrance; ^2^ Institut des Neurosciences de MontpellierINSERM U1051MontpellierFrance; ^3^ Université MontpellierMontpellierFrance; ^4^ Institut des Biomolecules Max Mousseron (IBMM)UMR5247‐CNRS‐UM ENSCM Faculté de PharmacieMontpellierFrance; ^5^ Centre de référence des affections sensorielles génétiquesCHRUMontpellierFrance

**Keywords:** all‐*trans*‐retinal, bisretinoid A2E, chromene adduct, phloroglucinol, photoreceptor, retinal pigment epithelium

## Abstract

Among retinal macular diseases, the juvenile recessive Stargardt disease and the age‐related degenerative disease arise from carbonyl and oxidative stresses (COS). Both stresses originate from an accumulation of all‐*trans*‐retinal (a*t* RAL) and are involved in bisretinoid formation by condensation of a*t* RAL with phosphatidylethanolamine (carbonyl stress) in the photoreceptor and its transformation into lipofuscin bisretinoids (oxidative stress) in the retinal pigment epithelium (RPE). As a*t* RAL and bisretinoid accumulation contribute to RPE and photoreceptor cell death, our goal is to select powerful chemical inhibitors of COS. Here, we describe that phloroglucinol, a natural phenolic compound having anti‐COS properties, protects both rat RPE and mouse photoreceptor primary cultures from a*t* RAL‐induced cell death and reduces hydrogen peroxide (H_2_O_2_)‐induced damage in RPE in a dose‐dependent manner. Mechanistic analyses demonstrate that the protective effect encompasses decrease in a*t* RAL‐induced intracellular reactive oxygen species and free a*t* RAL levels. Moreover, we show that phloroglucinol reacts with a*t* RAL to form a chromene adduct which prevents bisretinoid A2E synthesis *in vitro*. Taken together, these data show that the protective effect of phloroglucinol correlates with its ability to trap a*t* RAL and to prevent its further transformation into deleterious bisretinoids. Phloroglucinol might be a good basis to develop efficient therapeutic derivatives in the treatment of retinal macular diseases.

## Introduction

Major damage to the outer segment of post‐mitotic photoreceptors and in the adjacent retinal pigment epithelium (RPE) is caused by both carbonyl and oxidative stresses (COS) 1, 2, 3. Carbonyl stressors are reactive carbonyl species (RCS) such as naturally occurring aldehydes or produced by lipid peroxidation, and oxidative stress results from the generation of an excess of reactive oxygen species (ROS). Together, COS cause *in vivo* harmful macromolecular modifications (alkylations, peroxidations and hydroxylations) that participate in loss of function, structural disorganization and the appearance of intracellular material often fluorescent and resistance to degradation referred to as retinal age pigment or lipofuscin 4, 5. These changes, increasing progressively over time, together constitute an aging process, and, as a consequence, threaten survival of the retinal cells.

The RPE performs critical functions to maintain healthy photoreceptors including the regeneration of the visual retinoid chromophore 11‐*cis*‐retinal (11*c*RAL), and the ingestion and degradation of the photo‐oxidized apical tips of photoreceptor outer segments (POS) by phagocytosis 6. This phagocytosis process is itself an oxidative event 7, but the RPE scavenges the ROS by enzymatic or non‐enzymatic mechanisms, and as such could be considered as a barrier to oxidant effects 8.

Some of the RCS and ROS which are detrimental to photoreceptors and RPE originate from a*t*RAL accumulation following the photo‐isomerization process 9, 10. In the POS disc membranes, the visual chromophore 11*c*RAL is photo‐isomerized to a*t*RAL released from the light‐activated visual pigments 11. An active mechanism takes care of the clearance of a*t*RAL, preventing the toxicity that would otherwise be associated with the free aldehyde. The latter displays a high affinity for phosphatidylethanolamine (PE) and readily generates the Schiff base adduct N‐retinylidene‐PE (NRPE). NRPE is transported by ABCA4, a photoreceptor‐specific ATP‐binding cassette transporter, to the cytoplasm where it is expected to undergo hydrolysis to a*t*RAL and PE with subsequent reduction of a*t*RAL into non‐toxic all‐*trans‐*retinol by NADPH‐dependent RDHs 12, 13. A bright light exposure that bleaches substantial amounts of visual pigment will inevitably produce high (millimolar) concentrations of a*t*RAL, some of which could escape from reduction and generate acute toxicity in light‐induced photoreceptor degeneration 14. The mechanisms of this a*t*RAL toxicity include overproduction of ROS 10.

Most of the a*t*RAL is used to regenerate 11*c*RAL through the retinoid cycle, but a fraction of a*t*RAL can react non‐enzymatically with NRPE. This second condensation reaction leads to the formation of fluorescent bisretinoid compounds harmful to the RPE 15. The main bisretinoids currently described are the fluorophore A2E, a N‐retinylidene‐N‐retinylethanolamine and the retinal dimer. Both of them accumulate within RPE cell lysosomes upon ingestion of POS and their oxidation is accompanied by ROS formation and ultimately by their breaking down into RCS 16, 17. In Stargardt macular dystrophy, mutations in the *ABCA4* gene lead to accumulation of bisretinoids. 18. Mounting evidence also suggests that chronic oxidative stress may damage the RPE and predispose to the development of age‐related macular degeneration (AMD). 19. The RPE undergoes age‐dependent phagocytic and metabolic deficiency leading to retinal deposits called drusen, situated under the RPE of AMD patients, and comprising insoluble aggregates of oxidized lipids and proteins derived from the photochemical reactions in POS.

Based on epidemiology studies, natural antioxidants such as polyphenols appear as efficient protectors against oxidative stress. This activity may be related to their capacity to block the formation and accumulation of ROS, or to stimulate the enzymatic antioxidant defences of the organism. 20, 21. Recent literature addressed the efficiency of polyphenols to act as anti‐carbonyl stressor agents by trapping reactive toxic carbonyl entities. 22, 23. Phloroglucinol is a natural monomer of phlorotannins abundantly present in *Ecklonia cava* (edible brown algae). Phloroglucinol was shown to reduce oxidative damages in cell culture experiments and to react with glyoxal and methylglyoxal, contributing to the inhibitory effect of phlorotannins on the formation of advanced glycation end products 24, 25.

In this study, we wished to test the capacity of phloroglucinol to inhibit the formation of COS. We found that phloroglucinol protects both RPE and photoreceptors when challenged with a toxic dose of a*t*RAL and reduces H_2_O_2_‐induced damage in RPE. Furthermore, we show that it combines with a*t*RAL to form a chromene adduct, thus reducing the A2E synthesis. Our data demonstrate for the first time that phloroglucinol may counter the acute deleterious effect of a*t*RAL accumulation in the outer retina.

## Materials and methods

### Chemicals

Trypsin, collagenase, Dulbecco's modified eagle's medium (DMEM), DMEM/HamF12 and foetal bovine serum (FBS) were from Gibco (Paisley PA4 9RF, UK), and were used for RPE cell cultures. Phloroglucinol was purchased from Sigma‐Aldrich (St. Louis, MO, USA) and was dissolved in dimethylsulfoxide (DMSO) to prepare a stock solution at 10 mg/ml. The stock solution was diluted in culture medium to obtain final concentrations in 0.1% DMSO. a*t*RAL (Sigma‐Aldrich) was dissolved in dimethylfomamide (DMF) and diluted in serum‐free culture medium to final concentrations in 0.1% DMF. All the operations were carried out under dim red light. H_2_O_2_ (3%, Gifrer Barbezat, Décines, France) was diluted with serum‐free culture medium to final concentrations. 3‐(4, 5‐dimethylthiazol‐2‐yl)‐2, 5‐diphenyl tetrazolium bromide (MTT) was obtained from Sigma‐Aldrich and was used at 0,5 mg/ml. 2′,7′dichloro‐fluorescin diacetate (DCFDA) was from Abcam (Cambridge, UK).

### Animals

Long‐Evans rats were obtained from Depre (Saint‐Doulchard, France). Colony was maintained in an animal room, subjected to standard light cycles (12 hrs light and 12 hrs dark), and was fed *ad libitum* with a standard rodent diet. All experiments were performed in accordance with the Association for Research in Vision and Ophthalmology Statement for the Use of Animals in Ophthalmic and Vision Research and were approved by the local Ethics Committee. C57BL/6J mice were from JANVIER LABS (Saint‐Berthevin, France).

### Retina cell cultures

Primary rat RPE cells were established from Long‐Evans new‐born rats according to the procedure described elsewhere with modifications. 26 Briefly, eyes were enucleated and soaked overnight in the dark at room temperature (RT) in DMEM. The intact globes were then incubated for 60 min. at 37°C with 0.5 ml of trypsin–collagenase solution per eye (1 mg trypsin per ml and 2 mg collagenase per ml dissolved in DMEM). Immediately after incubation, the eyes were immersed in culture medium (DMEM, 10% (v/v) FBS, 1% (v/v) antibiotics). Intact sheets of RPE were then gently separated from the choroid, pooled, centrifuged at 100 *g* for 10 min., rinsed in phosphate buffer saline (PBS) and again centrifuged at 100 *g* for 10 min. The RPE sheets were then incubated at RT in a trypsin‐EDTA solution to obtain a suspension of single cells. The cells were then centrifuged at 100 *g* for 10 min., resuspended in culture medium and cultured in 96‐well plates for cell viability assays and ROS measurements or 24‐well plates for high‐performance liquid chromatography (HPLC) analysis. Cultures were maintained in an incubator in a 95% air/5% CO_2_ atmosphere at 37°C. After 3 days in culture, the primary rat RPE cells reached 80–85% of confluence. RPE cells were examined with a Zeiss phase contrast inverted microscope and photographed with a DP20 Olympus digital camera.

Mouse neural retina (NR) primary cultures were obtained from 3‐day C57BL/6J pups. Briefly, retina were dissected and digested by a papain (82.5 U)/DNase (2000 U) solution for 40 min. at 37°C before adding 1.5% of ovomucoïd to end papain. Dissociation of cells was obtained by transfer of cells in sterilized pipets slowly and drop wise. Cells were centrifuged at 830 r.p.m. for 6 min. and resuspended with 2/3 DMEM (Gibco) and 1/3 AmnioMAX‐C100 Supplement (Gibco), 10% FBS (Lonza) and 1% antibiotics (Lonza). The resulting cell suspension was seeded on 96‐well or 24‐well plates for cell viability or immunochemistry, respectively.

### Cell treatment

Pre‐ and co‐treatment procedures with phloroglucinol were carried out. During pre‐treatments, rat RPE primary cultures received a medium containing phloroglucinol at different concentrations (0.5–50 μg/ml) for 24 hrs. The medium was then removed and replaced by a serum‐free culture medium containing either a*t*RAL or H_2_O_2_ for 2 hrs. During co‐treatments, RPE and NR cells received a serum‐free medium containing phloroglucinol and either a*t*RAL or H_2_O_2_ for 4 hrs.

### Cell viability

Cell viability was determined by MTT assay. 27 Cells were incubated for 2 hrs with fresh culture medium containing 0.5 mg/ml MTT. During this incubation time, dehydrogenases of living cells reduced MTT to insoluble purple formazan, which was then dissolved. The absorbance at 570 nm of individual wells was measured by a microplate reader.

### ROS production

Reactive oxygen species (ROS) were measured in primary rat RPE cells with the probe DCFDA. The cell permeant reagent DCFDA is deacetylated by cellular esterases to dichlorofluorescein (DCFH), which can be oxidized by ROS into the fluorophore 2′, 7′ –dichlorofluorescein (DCF). Cells were seeded on white, opaque‐bottomed 96‐well plates. On day 3, the media were removed and the cells were washed with 1X Buffer (supplied with the kit) and incubated for 45 min. at 37°C in 1X Buffer containing 25 μM DCFDA. The cells were then washed with 1X Buffer and co‐incubated with phloroglucinol and a*t*RAL in 1X Buffer for 4 hrs at 37°C. DCF production was measured by fluorescence spectroscopy with excitation wavelength at 485 nm and emission wavelength at 535 nm.

### Immunocytochemistry

Primary mouse NR cells were fixed in 4% paraformaldehyde for 15 min. at room temperature, permeabilized in 0.1% triton, incubated with mouse monoclonal anti‐Rhodopsin antibody RET‐P1 (Novus Biologicals^®^, NBP120‐3267 diluted at 1:500) and revealed with Alexa594‐conjugated anti‐rabbit. Imaging of Rhodopsin‐immunoreactive (IR) cells was performed with a Zeiss AxioImager Z1 with ApoTome attachment. Images' acquisitions were obtained with the Zeiss Zen software and Rhodopsin‐IR cells were counted with Zen software.

### HPLC analysis

Following co‐treatment of primary rat RPE cells with a*t*RAL and phloroglucinol, medium was collected and cells were lysed in PBS with 0.2% SDS and frozen at −80°C until used. Retinal and derivatives were extracted with hexane under dim red light and resolved with a Varian HPLC system equipped with a reverse‐phase C18 Isis column (4.6 × 250 mm) (Macherey‐Nagel) and a Prostar 330 diode array detector. The elution was performed with 80% acetonitrile in water for 40 min. at a flow rate of 1 ml min.^−1^. They were analysed at specific absorption wavelengths of 298, 380 and 430 nm, and quantified from the peak areas by calibration curves determined with established standards.

### Synthesis of a major atRAL‐phloroglucinol adduct

Experimental conditions were carried out based on those previously reported to optimize the one‐step synthesis of A2E. 28 The reaction was performed with phloroglucinol and a*t*RAL in equimolar ratio, and in the presence of acetic acid (0.1 equivalent) and ethanol as solvent. Ethanol volume was determined to obtain a concentration of reactants between 0.01 and 0.1 M to promote the intermolecular reactions. The reaction was placed at room temperature in the dark and absence of air, under constant agitation, for a period of 1–4 days. Samples were taken regularly and analysed by HPLC. The elution was performed as aforementioned and detected at 298 and 380 nm.

### Elucidation of adduct chemical structure: the chromene

To fully characterize this adduct, its synthesis was performed in a mg scale so that enough material of adduct could be isolated after purification. Optimization of the reaction lead us to use one equivalent of phloroglucinol (0.35 mmol) for one equivalent of a*t*RAL and the reaction was catalysed by one equivalent of acetic acid in ethanol (8 ml). The reaction was stirred at room temperature for 48 hrs, protected from the light with foil paper. After concentration of solvent under reduced pressure, the residue was dissolved in ethyl acetate (20 ml) and washed with water (10 ml). The organic layer was recovered, dried on MgSO_4_ and concentrated under reduced pressure. The residue obtained was purified by chromatography on silica gel (9/1 to 8.5/1.5 pentane/ethyl acetate) followed by preparative HPLC (*t*_0′_ = 0/10, *t*_15′_ = 90/10_,_ *t*_45′_ = 80/20_,_ *t*_75′_ = 70/10_;_ Hexane/ethyl acetate, 15 mL/min., column luna 5μ Silica 100Å 250 × 21.20 mm, detection 254 nm) to give rise to the major adduct as a purple solid. Chemical structure of the isolated compound was determined by NMR ^1^H and ^13^C (performed with HMBC and HSQC correlation between ^1^H and ^13^C atoms) and by mass spectrometry (MS) analysis.

### Synthesis of A2E and competition reaction

A2E was synthesized as described previously 28 from ethanolamine and a*t*RAL in a 1:2 ratio. The product was characterized by HPLC retention time and UV‐visible absorption spectra. The data were in agreement with those reported. Competition between phloroglucinol and ethanolamine was conducted with two and one equivalent, respectively, per two equivalent a*t*RAL and one equivalent acetic acid. The reaction was placed at room temperature in the dark, under constant agitation, for a period of 3 days. Samples were analysed by HPLC and synthesis of A2E and adduct was monitored at 430 and 298 nm, respectively.

### Statistical analysis

The data are presented as means ± SEM determined from at least three independent experiments. In each experiment, all conditions were done at least in triplicate. Statistical analysis was performed by *t*‐test with normal distribution and differences with *P* values <0.05 were considered as statistically significant.

## Results

### Cytotoxic effects of a*t*RAL and H_2_O_2_ on RPE cells

**Figure 1:**
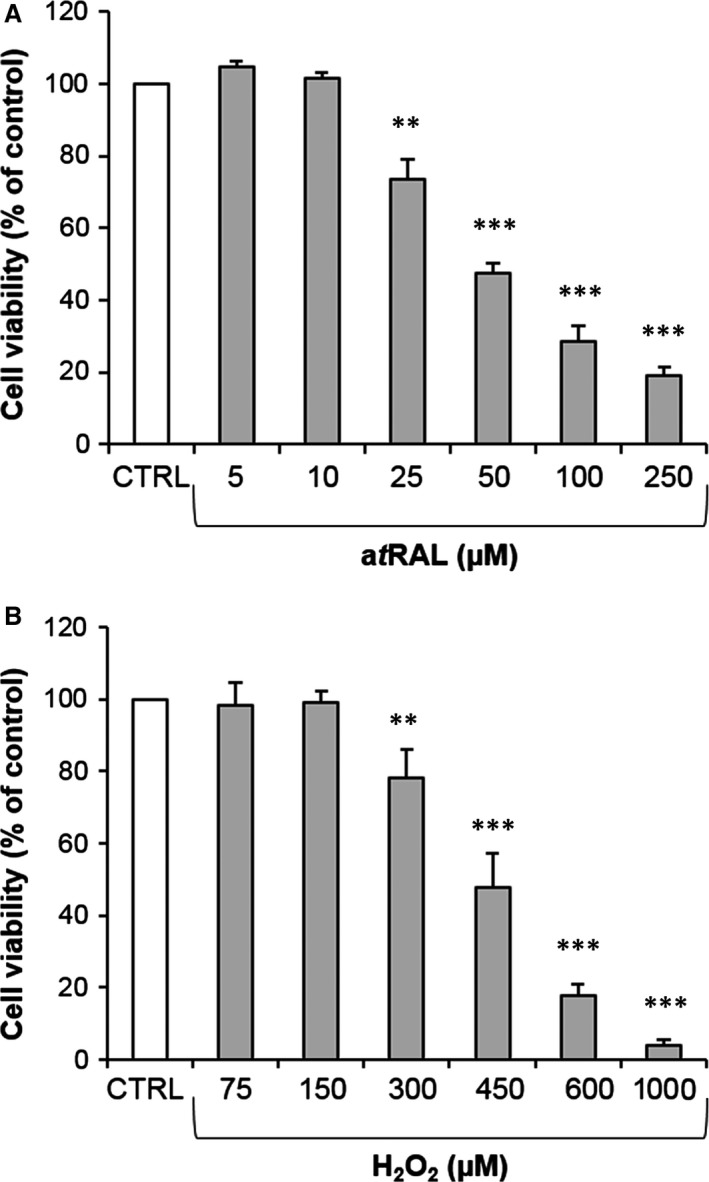
Primary rat RPE cell death induced by a*t* RAL and H_2_O_2_. (**A**,** B**) Primary rat RPE cells were incubated in serum‐free medium with a*t* RAL (**A**) or H_2_O_2_ (**B**) for 2 hrs at various concentrations as indicated. Cell viability was determined by MTT assay. The data are expressed as the percentage of control untreated cells and presented as means ± SEM (*n* = 3 independent experiments, each condition at least in triplicate). **P < 0.01, ***P < 0.001 *versus* untreated, *t*‐test. RPE, retinal pigment epithelium.

To choose the appropriate concentrations of stressors that caused significant cell death, we first performed dose–response assays. As shown in Figure 1A, treatment of primary rat RPE cells with a*t*RAL caused a dose‐dependent decrease in cell viability with an IC50 of around 50 μM. Treatment with H_2_O_2_ resulted in cell viability loss with an IC50 close to 450 μM (Fig. 1B) as previously reported 29. Therefore, concentrations of 50 μM a*t*RAL and 450 μM H_2_O_2_ were initially used as the working concentrations.

### Pre‐treatment of RPE cells with phloroglucinol protects from a*t*RAL‐ and H_2_O_2_‐ induced damage

**Figure 2:**
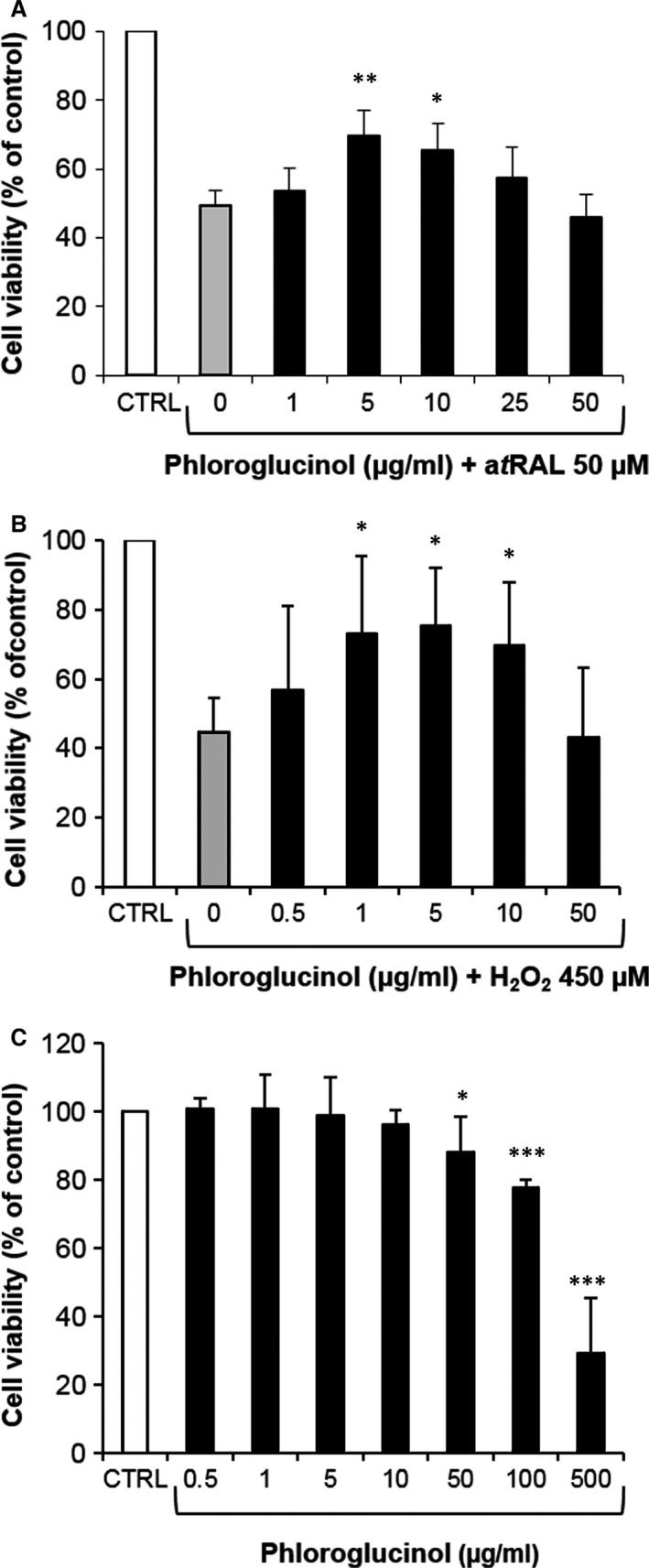
Pre‐treatment of RPE cells with phloroglucinol inhibits carbonyl and oxidative stresses‐induced cell death. (**A**,** B**) Primary rat RPE cells were pre‐treated with increased concentrations of phloroglucinol for 24 hrs, washed and exposed to 50 μM a*t* RAL (**A**) or 450 μM H_2_O_2_ (**B**) for 2 hrs. (**C**) RPE cells were incubated for 24 hrs with various concentrations of phloroglucinol. Cell viability was determined by MTT assay. The data are expressed as the percentage of control untreated cells and presented as means ± SEM (*n* = 4–5 independent experiments, each condition at least in triplicate). *P < 0.05, **P < 0.01, ***P < 0.001 *versus* untreated, *t*‐test. RPE, retinal pigment epithelium.

Pre‐treatment with phloroglucinol was carried out to investigate its cytoprotective effect on COS within primary rat RPE cells. First, RPE cells were pre‐treated for 24 hrs with various concentrations of phloroglucinol (1–50 μg/ml), rinsed and incubated with 50 μM a*t*RAL for 2 hrs. RPE cells incubated with a*t*RAL displayed 49 ± 5% cell viability (Fig. 2A), whereas pre‐treatment with 5 or 10 μg/ml (40 or 80 μM) phloroglucinol before exposure to a*t*RAL provided significant increases in cell viability (70 ± 7% and 66 ± 8%, respectively). Second, we assessed the anti‐oxidative effect of phloroglucinol. The treatment of RPE cells with 450 μM H_2_O_2_ resulted in 45 ± 10% viability (Fig. 2B), whereas pre‐treatment with phloroglucinol at 1, 5 or 10 μg/ml before H_2_O_2_ resulted in 73 ± 22%, 76 ± 16% and 69 ± 18% of cell viability, respectively. Thus, pre‐treatment of RPE cells with phloroglucinol provides protection from both a*t*RAL and H_2_O_2_‐mediated toxicity with similar effectiveness. Above 10 μg/ml phloroglucinol, we also found significantly less protection, which was shown by the biphasic survival curves (Fig. 2A,B). This phenomenon could be attributed to the cytotoxicity of phloroglucinol at concentrations higher than 10 μg/ml (Fig. 2C) as previously reported on neuronal cells 24, 30. Indeed, 50, 100 and 500 μg/ml resulted in reduction in cell viability (88 ± 10%, 77 ± 3% and 29 ± 16%, respectively).

### Co‐treatment with phloroglucinol and a*t*RAL protects RPE cells

**Figure 3:**
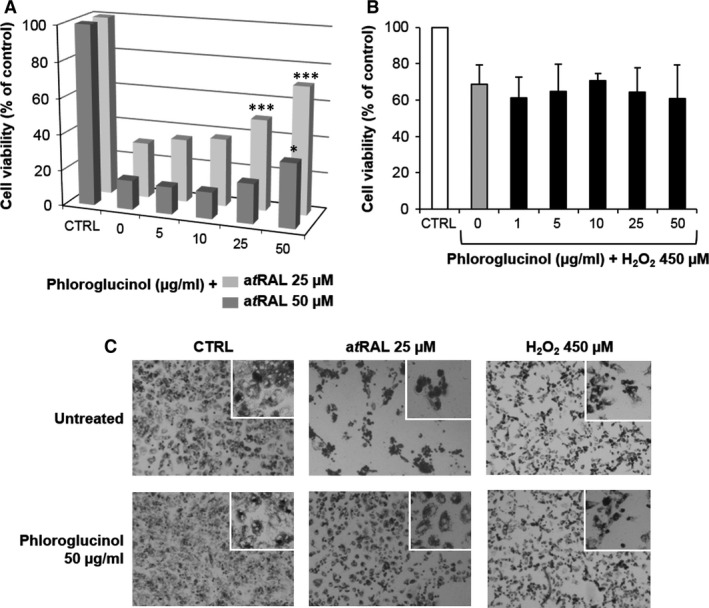
Co‐treatment with phloroglucinol and a*t* RAL protects RPE cells. (**A** and **B**) RPE cells were co‐incubated with increased concentrations of phloroglucinol and either 25 or 50 μM a*t* RAL (**A**), or 450 μM H_2_O_2_ (**B**), for 4 hrs. Cell viability was determined by MTT assay. The data are expressed as the percentage of control untreated cells and presented as means ± SEM (*n* = 4–5 independent experiments, each condition at least in triplicate). *P < 0.05, **P < 0.01, ***P < 0.001 *versus* untreated, *t*‐test. (**C**) Photographs show a good preservation of RPE cell morphology with 50 μg/ml phloroglucinol in the presence of 25 μM a*t* RAL but not with 450 μM H_2_O_2_. Higher magnification (inserts) shows that RPE cells treated with a*t* RAL were rounded and compacted. In contrast, cells co‐treated with phloroglucinol kept a polygonal morphology. Treatment with H_2_O_2_ caused the RPE cells to deform and shrink. However, co‐treatment with phloroglucinol did not attenuate these morphological changes. RPE, retinal pigment epithelium.

We next assessed the capacity of phloroglucinol to protect RPE during co‐incubation for 4 hrs, a condition to probe its scavenging properties. RPE cells were treated with phloroglucinol (5–50 μg/ml) in the presence of a*t*RAL (25–50 μM) or H_2_O_2_ (450 μM). Co‐treatment of RPE cells with 50 μg/ml phloroglucinol significantly reduced the toxic effect of 25 μM a*t*RAL (71 ± 13% and 31 ± 3% of cell viability, respectively) and 50 μM a*t*RAL (36 ± 10% and 16 ± 2% of cell viability) (Fig. 3A). In contrast, phloroglucinol had no protective effect on H_2_O_2_ toxicity (Fig. 3B,C). In the same way, images of primary rat RPE cells co‐treated with phloroglucinol (50 μg/ml) and a*t*RAL (25 μM) for 4 hrs clearly showed a good preservation of cell morphology comparable to untreated cells and in contrast to the marked cell death induced by a*t*RAL (Fig. 3C). Indeed, the RPE cells treated with a*t*RAL were rounded and compacted, with a loss of cell adhesion. In contrast, most cells co‐treated with phloroglucinol and a*t*RAL kept a characteristic polygonal morphology. Treatment with H_2_O_2_ caused the RPE cells to deform and shrink. However, phloroglucinol did not attenuate these morphological changes. Thus, co‐treatment of primary rat RPE cells with phloroglucinol provides a protection against a*t*RAL but not H_2_O_2_.

### Phloroglucinol protects photoreceptor cells from a*t*RAL damage

**Figure 4:**
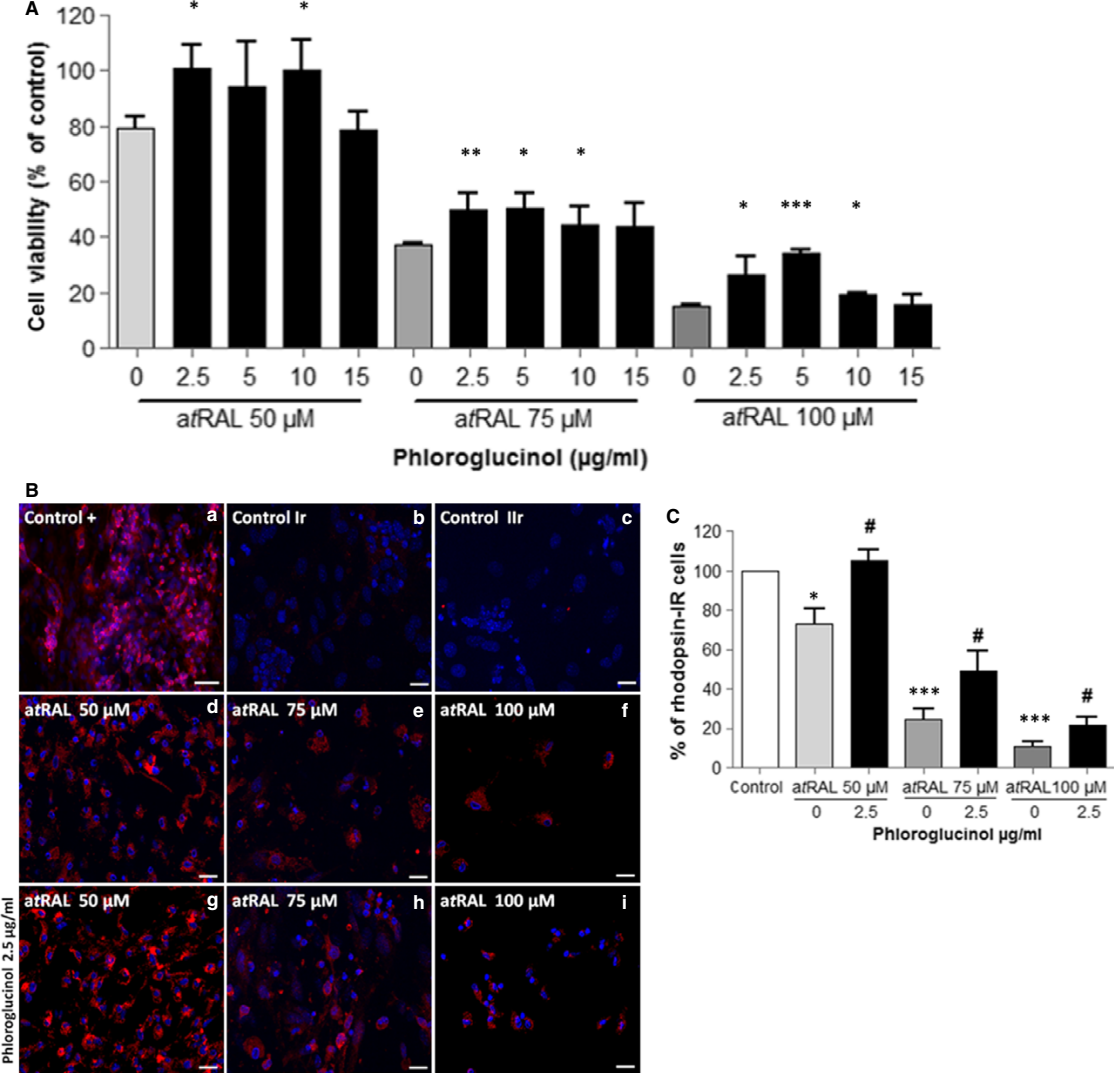
Phloroglucinol protects primary mouse photoreceptor cells against a*t* RAL. (**A**) Primary mouse neural retina cultures were treated for 4 hrs with various concentrations of a*t* RAL as indicated (grey bars) or co‐treated with increased concentrations of phloroglucinol (black bars). After 18 hrs in a fresh medium, cell viability was determined by MTT assay. (**B**) Alternatively, cells were fixed, permeabilized, incubated with Rhodopsin antibody, and revealed with Alexa 594‐conjugate secondary antibody. Control +: untreated cultures; control Ir and control IIr: incubation without primary and secondary antibody, respectively. (**C**) Rhodopsin‐IR cells were counted. The data are expressed as the percentage of control untreated cells presented as means ± SEM (*n* = 3 independent experiments, each condition at least in quintuplicate). *P < 0.05, **P < 0.01, ***P < 0.001 *versus* untreated, *t*‐test. **#** *P* < 0.05, *versus* a*t* RAL‐treated cells.

As photoreceptor cells are the site of photo‐isomerization of 11*c*RAL to a*t*RAL and may accumulate a*t*RAL in pathological circumstances 31, we investigated the damaging effect of a*t*RAL in mouse neural retina primary cultures as well as the protective action of phloroglucinol. Ten days *in vitro* retinal cultures were treated with different concentrations of a*t*RAL and showed a dose‐dependent cell death (Fig. 4A, grey bars). Co‐incubation of phloroglucinol with a*t*RAL increased the cell survival in a concentration‐dependent manner compared with a*t*RAL treatment alone (Fig. 4A, black bars). Protection of photoreceptor cells was confirmed with imaging and cell counts after immunochemistry with Rhodopsin antibodies (Fig. 4B). In control cultures, we counted the same amount of Rhodopsin‐IR positive and negative cells. The number of Rhodopsin‐IR positive cells was significantly higher in cell cultures incubated with 2.5 μg/ml phloroglucinol relative to samples exposed to a*t*RAL alone (Fig. 4C). When the culture was exposed to a*t*RAL and phloroglucinol, we obtained 71 ± 9% of Rhodopsin‐IR–positive cells. This occurred with more protection of photoreceptors by phloroglucinol and a higher toxicity of a*t*RAL on Rhodopsin‐IR–negative cells. We inferred that the protective effect of phloroglucinol against a*t*RAL was not only confined to RPE but also applied to the photoreceptors.

### Mechanisms of the anti‐COS effect of phloroglucinol

To advance in our understanding of the mechanisms involved in its protective effects, we hypothesized that phloroglucinol could both prevent oxidative stress by scavenging intracellular ROS and carbonyl stress by trapping free a*t*RAL.

**Figure 5:**
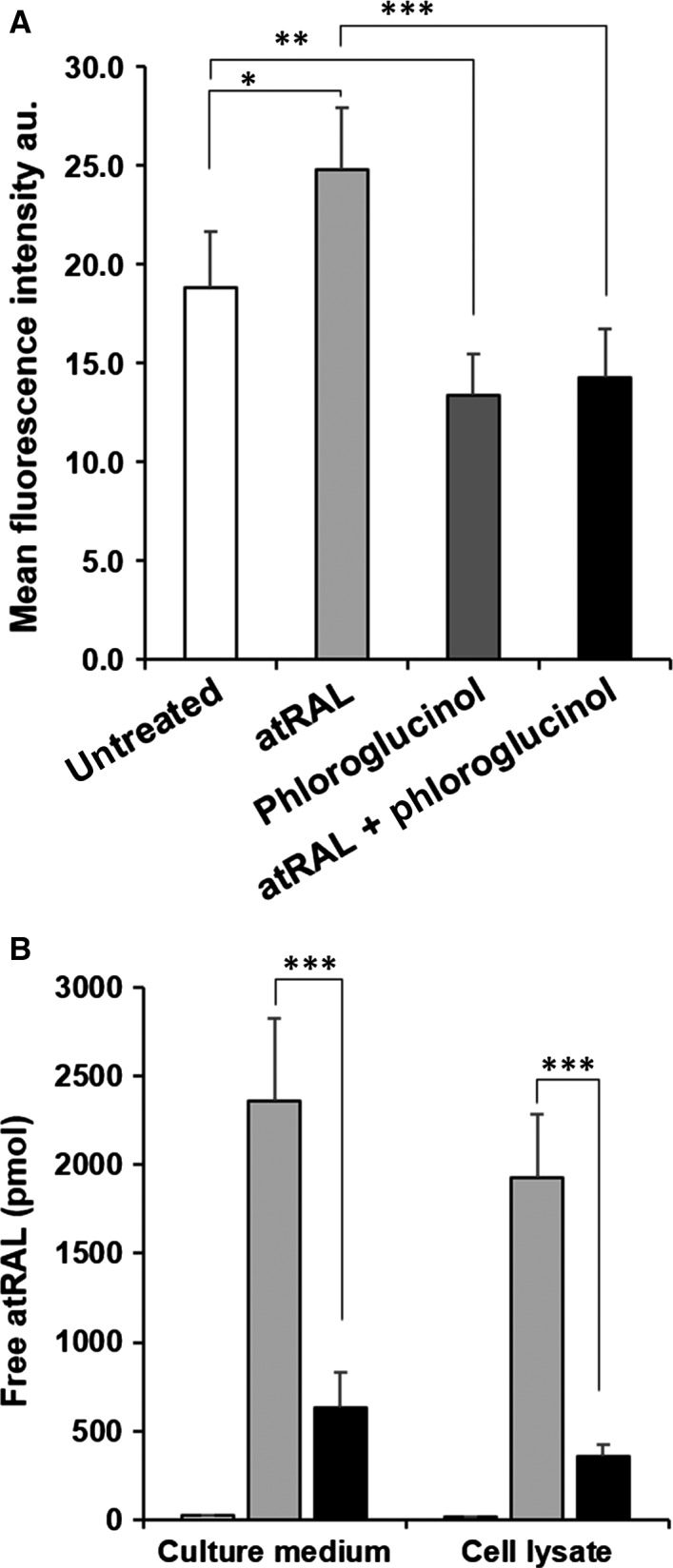
Phloroglucinol prevents a*t* RAL‐induced ROS production and traps a*t* RAL in primary rat RPE. (**A**) Primary rat RPE cells were incubated with DCFDA for 45 min. and then treated with 25 μM a*t* RAL alone (grey squares) or with 50 μg/ml phloroglucinol (black squares) for additional 4 hrs. Fluorescent intensity was measured at λ_em_ 535 nm (λ_exc_ = 485 nm) and expressed as arbitrary units. The data are expressed as means ± SEM (*n* = 4 independent experiments, each condition at least in triplicate). *P < 0.05, **P < 0.01, ***P < 0.001 *versus* untreated, *t‐*test. (**B**) Similar RPE treatments were performed and retinoids were extracted from culture media and cell lysates. The free a*t* RAL content (pmol) was quantified by HPLC from standard calibration. Traces of a*t* RAL were detected in DMEM:F‐12 medium (white squares). The data are from a representative experiment repeated three times and presented as means ± SD of sextuplicates. ***P < 0.001, *t‐*test. RPE, retinal pigment epithelium.

The radical scavenging effect of phloroglucinol on the intracellular ROS was measured with DCFDA as a probe of ROS levels. DCFDA‐loaded primary rat RPE cells were treated with 25 μM a*t*RAL and 50 μg/ml phloroglucinol for 4 hrs. As shown in Figure 5A, treatment of RPE cells with a*t*RAL alone enhanced the fluorescence intensity. On the contrary, co‐treatment with phloroglucinol reduced the fluorescence intensity reflecting a reduction in ROS generation.

We next incubated primary rat RPE cells with 25 μM a*t*RAL and 50 μg/ml phloroglucinol for 4 hrs before analysing free a*t*RAL both in the culture medium and the cell compartment (Fig. 5B). Without addition of a*t*RAL and phloroglucinol (DMEM/F12), a very small amount of free a*t*RAL was detected within the culture medium and the cell lysate. The addition of 25 μM a*t*RAL was quantitatively measured in both compartments (2360 ± 465 and 1926 ± 361 pmol in the culture medium and cell lysate, respectively). Upon co‐incubation with phloroglucinol, the amount of a*t*RAL was strikingly reduced (632 ± 200 and 359 ± 62 pmol, respectively). These results demonstrate that phloroglucinol reduces much of a*t*RAL, which could be explained by a trapping effect.

**Figure 6:**
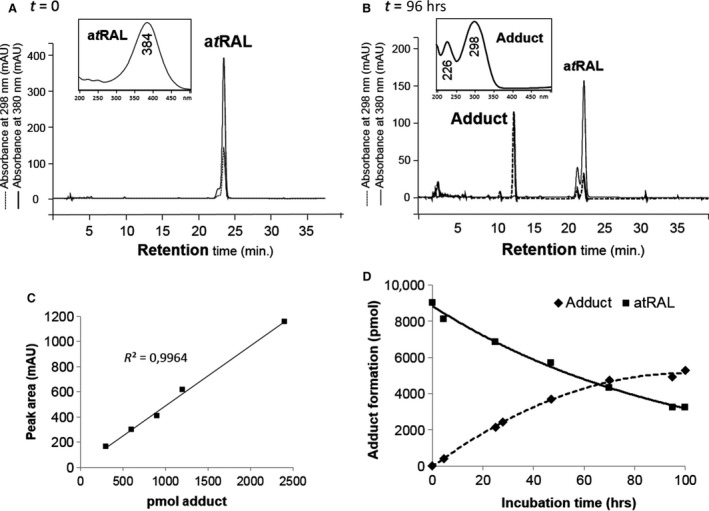
Phloroglucinol reacts with a*t* RAL to form chromene. Reverse‐phase HPLC chromatograms of an equimolar a*t* RAL/phloroglucinol mixture at the beginning (**A**,* t* = 0) and the end of the reaction (**B**,* t* = 96 hrs). Retention times and absorbance spectra (insets) of major peaks were used to identify the retinoid. Absorbance was then measured at 380 and 298 nm (λ_max_ of a*t* RAL and the major adduct, respectively). Adduct was purified by chromatography and isolated by HPLC to establish a linear calibration curve (**C**) and its absolute quantitation during its kinetic of formation (**D**). HPLC, high‐performance liquid chromatography.

To validate the trapping of a*t*RAL by phloroglucinol and characterize reaction products, we conducted *in vitro* reactions between a*t*RAL and phloroglucinol in acidic catalysis (as described in Materials and methods). Starting from a*t*RAL (Fig. 6A) and phloroglucinol (proportion 1/1) we identified the formation of a major product, which we described as ‘adduct’ (Fig. 6B). This adduct has an approximate retention time of 13.6 min. under the HPLC conditions, with absorption maxima λ_max_ at 226 and 298 nm. We established a calibration curve to quantify the relative amounts of this adduct formed during the time course (Fig. 6C). Phloroglucinol and a*t*RAL in an equimolar ratio yield large amount of adduct, which represents nearly 60% of total compounds (Fig. 6D).

### Molecular characterization of chromene adduct

**Figure 7:**
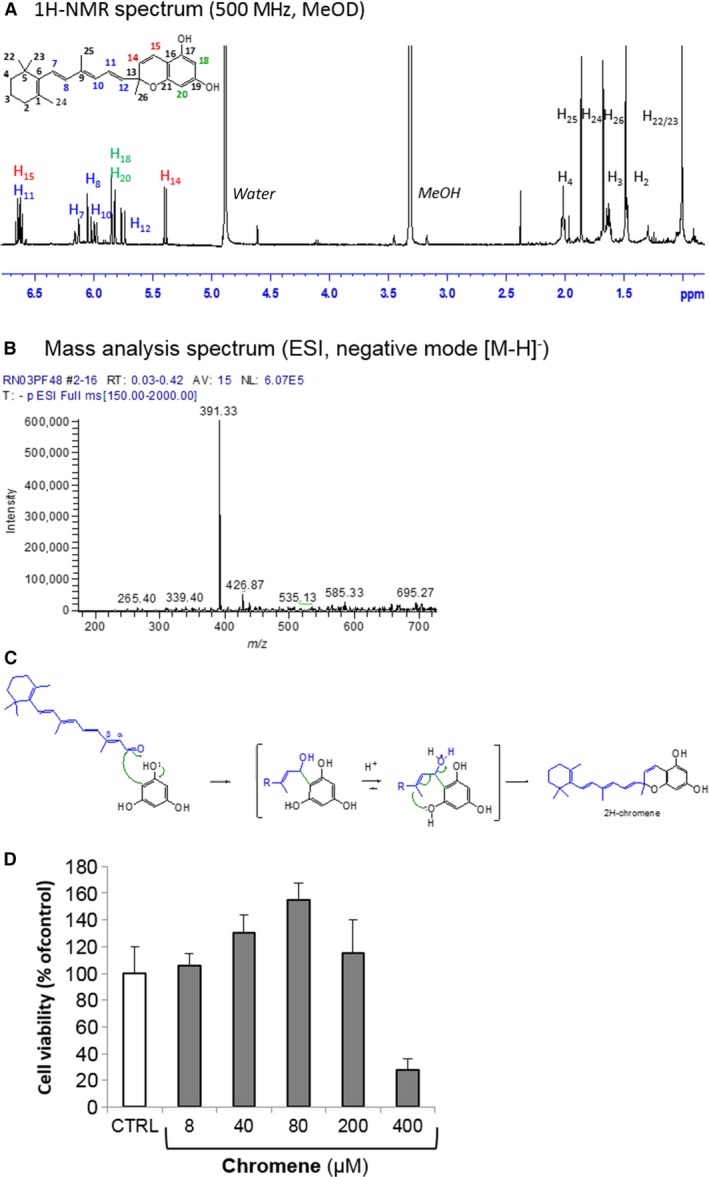
Structural characterization of the chromene adduct. Representative ^1^H‐NMR (**A**) and MS spectra (**B**) of the 2H‐chromene adduct are shown. ^1^H NMR (500 MHz) and *J‐*modulated ^13^C NMR (125 MHz) spectra were obtained with CDC1_3_ as solvent on a Bruker AVANCE II spectrometer. Chemical shifts are given in ppm with the solvent peaks for CDCl_3_ at δ_H_ 7.23, δ_C_ 77 ppm, respectively. Coupling constants are reported in Hz. MS analysis was performed with LCQ Advantage mass spectrometer. ESI‐MS was recorded on positive mode. (**C**) This 2H‐chromene is the result of first, a 1, 2‐*C*‐addition of the free carbon atom of the resorcinol framework onto the carbonyl group of a*t* RAL, followed by an intramolecular *O*‐addition onto the α‐β double bond. (**D**) Retinal pigment epithelium (RPE) cells were incubated for 24 hrs with various concentrations of chromene. Cell viability was determined by MTT assay. The data are from a representative experiment repeated two times and presented as means ± SD of sextuplicates.

This adduct was chemically synthesized in large mg scale, purified and analysed. Mass analysis, ^1^H and ^13^C NMR spectrum confirmed the chemical structure of the adduct and the presence of a chromene moiety (Fig. 7A,B). The mechanism of the chromene formation results in both C and O alkylations on the carbonyl function and on the first conjugated double bond of the a*t*RAL, respectively (Fig 7C). The elucidation of the chemical structure of the adduct formed during the reaction of phloroglucinol with a*t*RAL in our conditions, showed that nucleophilicity of the carbon atoms of phloroglucinol aromatic ring allows them to be reactive towards the a*t*RAL carbonyl electrophiles according to the HSAB theory 32. Respectively, the nucleophilicity of the phenoxide anion of the phloroglucinol is also adapted to react with a*t*RAL‐conjugated double bonds and thus stabilizes the adduct by the formation of chromene ring. Surprisingly, this chromene appears to improve the survival of primary rat RPE cells at concentrations at least up to 80 μM (Fig. 7D). This protection was related to an anti‐oxidant effect of this adduct compound (Fig. S1).

### Phloroglucinol inhibits A2E formation

**Figure 8:**
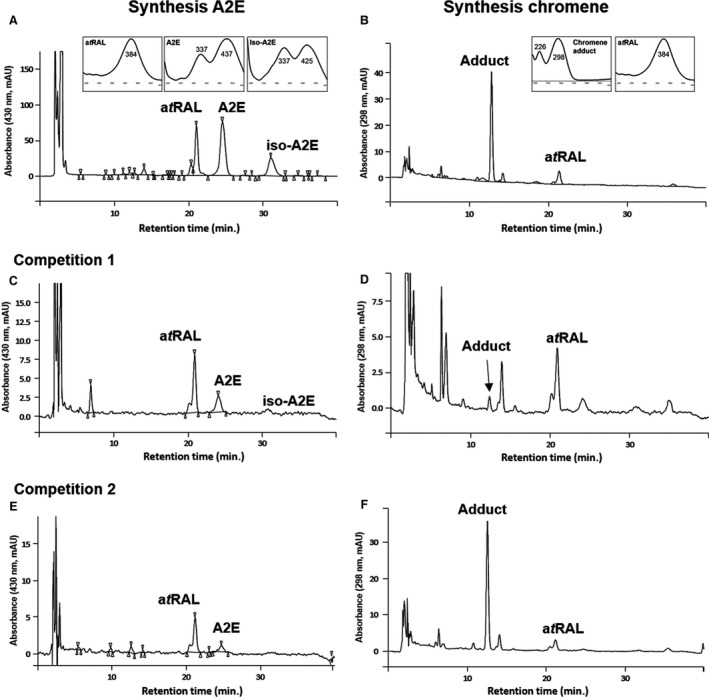
Reverse‐phase C18 HPLC monitoring of synthesis of A2E and chromene adduct. (**A**) The biomimetic synthesis of A2E was performed as previously described 28 with a*t* RAL (two equivalents) and ethanolamine (one equivalent) and followed at 430 nm. (**B**) The synthesis of chromene adduct required a*t* RAL (one equivalent) and phloroglucinol (one equivalent) and was quantified at 298 nm. Insets are UV‐visible absorbance spectra of A2E, iso‐A2E and chromene adduct. A2E exhibits absorbance maxima at 437 and 337 nm, iso‐A2E at 425 and 337 nm and chromene adduct at 298 and 226 nm. Competition with 1 (**C, D**) or 2 (**E, F**) equivalents phloroglucinol decreased both a*t* RAL and A2E. The optimal formation of adduct required a*t* RAL and phloroglucinol in equimolar ratio (**F)**.

Following these observations on phloroglucinol reactivity, competition experiments were performed to study the trapping action of phloroglucinol in the presence of ethanolamine, a condition that leads to A2E formation (Fig. 8, Table 1). Synthesis of A2E, iso‐A2E, a C13‐C14 Z‐isomer of A2E and the chromene adduct were monitored by reverse‐phase HPLC (Fig. 8) and characterized by UV‐visible absorbance spectra (Fig. 8 A, B absorbance spectra insets). A competition reaction was first conducted with equimolar amount of ethanolamine and phloroglucinol, and two‐fold excess of a*t*RAL (competition 1). The synthesis of A2E was considerably reduced, however, the chromene adduct was weakly present in the sample. This could be explained by the possible formation of other adducts that were not identified at 298 nm. We performed again competition 1 reaction and compared it with A2E synthesis by UPLC‐UV (200–800 nm) – Mass Spectrometry (MS) detection. (Fig. S2). We clearly identified A2E compound in mass spectrometry (peak at 34 min., m/z 592) and observed the reduction of A2E formation in the presence of phloroglucinol. As we already synthesized chromene, we were also able to find traces of this compound in competition 1 sample (peak at 9.88 min., m/z 393). Unfortunately there was no major peak identified either by UV or MS analysis during this competition experiment. It could be some mixed products formed in small amount and thus difficult to identify. Indeed, bearing three nucleophilic positions on its aromatic ring, the phlorogucinol would be able to trap two retinal moieties leading to the formation of dimeric chromene compounds. Another explanation would be the formation of less stable compounds, obtained from O or C alkylation, but not undergoing the final cyclization involved in the formation of the stable chromene ring. Another competition reaction was performed with an excess of phloroglucinol compared to ethanolamine (2/1), and equimolar amounts compared to a*t*RAL (competition 2). The synthesis of A2E was inhibited in favour of the formation of the chromene adduct (compare A2E synthesis and competition 2). This result suggests that the carbonyl electrophile function of the a*t*RAL is preferentially attacked by phloroglucinol compared to ethanolamine, leading to a significant reduction in A2E production during the synthesis.

**Table 1:** Phloroglucinol reduces the synthesis of A2E

Chemical reaction	Phloroglucinol equivalent	A2E nmol	Chromene nmol
A2E synthesis	0	1833 ± 303	0
Competition 1	1	49 ± 19	10 ± 4
Competition 2	2	8 ± 7	318 ± 25

All chemical reactions (200 μL) were performed with a*t*RAL (2 mg, 7.0 μmol, 2 equivalents), ethanolamine (one equivalent) and acetic acid (one equivalent) in ethanol. A2E synthesis was done in the absence of phloroglucinol. Competition 1 and 2 were performed in the presence of one or two phloroglucinol equivalent, respectively. A2E and chromene were analysed by HPLC and quantified by comparing the sample peak area (see Fig. 8) to a calibration curve. Quantitative values of A2E and chromene produced were expressed in nmol/reaction. Each reaction was performed in triplicate (means ± SEM).

## Discussion

Oxidative and carbonyl stress contribute to RPE and photoreceptor degeneration in conditions such as AMD and Stargardt disease. Here, we provide for the first time compelling evidence that phloroglucinol protects RPE and photoreceptors against a*t*RAL‐induced cytotoxicity.

In this work, we used primary cultures of RPE and photoreceptors rather than commonly used cell lines such as ARPE‐19 and 661W. Cell lines may have abnormal characteristics and respond differently to oxidative challenge compared with primary cells 33. Cells in primary culture are likely to reflect *in vivo* cell morphology and function more accurately. In our study, phloroglucinol shows a significantly greater protective effect in primary rat RPE than in ARPE‐19 34. Hanneken and co‐authors also showed that many flavonoids were more effective at protecting primary human RPE cells compared to the ARPE‐19 cell line 20. We also assessed phloroglucinol on a*t*RAL‐treated 661W cells and observed no protective effect at concentrations up to 10 μg/ml (data not shown). By contrast, primary mouse photoreceptors were protected against a*t*RAL at phloroglucinol concentration as low as 2.5 μg/ml. Similarly, Hanneken and co‐authors reported that some flavonoids that had no effect in ARPE‐19 were effective in primary human RPE 20. This difference might reflect a failure of phloroglucinol uptake or of the intracellular signalling pathways in the cell line. Further investigations will be therefore needed on the signalling pathway triggered by phloroglucinol in the RPE. Whatever the exact mechanism, the choice of retina primary cultures of retina was necessary to highlight the protective effect of phloroglucinol.

Phloroglucinol compounds have a wide range of clinical applications and are used as anti‐spasmodic, anti‐microbial, anti‐inflammatory and neuroregenerative medications among others; and they appear to be less toxic than other agents 35. Phloroglucinol can exert protective effects against oxidative stress‐induced cytotoxicity *in vitro* and *in vivo*. 24, 36, 37 As examples, phloroglucinol was recently reported to attenuate motor functional and cognitive deficits in animal models of Parkinson's and Alzheimer's diseases 30, 38. This neuroprotective effect was mainly caused by its antioxidative activity, both preventing the increase in intracellular ROS and the loss of expression of antioxidant enzymes. Oxidative mechanisms in RPE or NR involved in the formation of ROS and anti‐oxidant enzymes were previously documented 10, 14. Photo‐excitation of a*t*RAL generates ROS *via* an NADPH oxidase pathway in the retina of Abca4‐/‐Rdh8‐/‐ mice after bright light exposure and in cultured ARPE‐19 cells. Therefore, a*t*RAL acts as a potent generator of oxidative stress when it accumulates. Moreover, induction of phase 2 oxidoreductase genes protects RPE cells against retinaldehyde‐mediated photo‐oxidative damage 39. Meanwhile, H_2_O_2_ can be increased in RPE during phagocytosis of POS and generates catalase activity essential for protecting RPE cell against ROS 7. In our study, pre‐treatment with phloroglucinol protects RPE and PR against toxic doses of a*t*RAL and RPE against H_2_O_2_‐induced death. This protective effect was dose dependent with a maximum (20–30% of cell survival gain) in a concentration range from 2.5 to 10 μg/ml and it decreased at higher concentrations. Similar dose responses and biphasing responses were previously reported in human cell lines, suggesting the implementation of related protective mechanisms and a cell toxicity of phloroglucinol at high doses 24, 30, 37, 38, 40. These protective mechanisms likely encompass the scavenging effect against ROS, as demonstrated by the decrease in a*t*RAL‐induced ROS production in RPE during co‐incubation with phloroglucinol (Fig. 5). Our data agree with previous reports on the radical scavenging effects of phloroglucinol 24, 41, 42.

Regarding the protective effect against a*t*RAL, a scavenging mechanism also correlates well with the decrease in free a*t*RAL seen both in the culture medium and cells during the co‐incubation of RPE with phloroglucinol, suggesting that the protection was at least related to the trapping of a*t*RAL. RPE cells co‐treated with phloroglucinol and a*t*RAL also showed a better preservation of cell morphology than in phloroglucinol pre‐treatments, supporting the hypothesis that in these conditions phloroglucinol trapped a*t*RAL before it could exert its effects in the cell. Another distinction between pre‐ and co‐treatment is that the maximum protection was observed for higher concentrations of phloroglucinol in co‐treatment (25–50 μg/ml) than in pre‐treatment experiments (2.5–10 μg/ml). This observation can be explained by the need of excess phloroglucinol to react with a*t*RAL in the culture condition. This differs from the equimolar ratio in the chemical synthesis of chromene made in acidic catalysis. However, we observed that this reduction in free a*t*RAL took place only in the presence of cells, probably involving an acidic intracellular compartment yet to be defined. On the contrary, the protection against H_2_O_2_ observed with pre‐treatment but not with co‐treatment rules out the possibility of pholoroglucinol acting directly against H_2_O_2_.

To confirm the supposed trapping mechanism with a*t*RAL, we conducted a series of chemical experiments. We showed that in equimolar proportions phloroglucinol was able to trap a*t*RAL through a double C and O alkylation leading to a stable chromene adduct. This chromene was devoid of cytotoxicity at high concentration up to 200 μM. The stability brought to the formation of a chromene cycle makes phloroglucinol an efficient agent to trap reactive carbonyls. Other reports have described the scavenging capacity of phloroglucinol for reactive carbonyl species under physiological conditions 23, 43. In this regard, α, β‐unsaturated aldehydes such as 4‐hydroxy‐*trans*‐2‐nonenal are produced by lipid peroxidation in PR, and dicarbonyls, such as glyoxal and methylglyoxal known to form advanced glycation end products (AGEs), are released upon photodegradation of A2E and all‐*trans*‐retinal dimer, two bisretinoids that accumulate as lipofuscin in the RPE. 17 We also noticed that depending on the stoichiometry between phloroglucinol and a*t*RAL, other not identified adducts would be formed. This result could explain that despite depletion of a*t*RAL in primary cells treated with phloroglucinol, the chromene derivative was not detected, suggesting the formation of different adducts in the cellular context (dimeric chromene compounds or reversible adducts). To support this hypothesis, it has been shown that a molar excess of phloroglucinol can form various adducts with glyoxal and methylglyoxal 25.

The potency of phloroglucinol was confirmed by its higher reactivity with a*t*RAL in the presence of ethanolamine leading to A2E synthesis inhibition. In the photoreceptors, free a*t*RAL can react *via* a Schiff base linkage with primary amines present in membrane phospholipids by a combination of carbonyl and oxidative stress 34, 44. The reactions with phosphatidylethanolamine can promote the formation of bisretinoids including A2E. The reactivity of phloroglucinol with respect to free a*t*RAL may therefore be of considerable benefit in reducing the amount of bisretinoid product over time and preventing the pathological mechanisms involved in Stargardt disease and AMD. However, if it occurs in the human retina, the irreversible trapping should be modulated so as to trap the free retinal in excess without affecting the retinoid cycle. Primary amines have previously been shown to be efficient by reacting with a*t*RAL without affecting the retinoid cycle 45.

Together, these data demonstrate that phloroglucinol has cytoprotective effects in outer retinal cells by scavenging ROS and trapping a*t*RAL. These effects may be extrapolated to prevent or ameliorate retinal function in patients suffering macular dystrophies, by selectively targeting retinaldehyde accumulation in the photoreceptor and improving RPE cell anti‐oxidative defences. A major disadvantage of phloroglucinol is its poor lipid solubility and low bioavailibility in the central nervous system. The results presented here should encourage the development of efficient therapeutic derivatives with improved selectivity for the retina.

## Conflict of interest

The authors confirm that there are no conflicts of interest.

## Supporting information


**Figure S1.** Protective and anti‐oxidant effects of chromene on rat primary RPE.

**Figure S2.** Comparaison of A2E synthesis reaction and competition 1 reaction.
