# Supplementary figures and images for: Phloroglucinol protects retinal pigment epithelium and photoreceptor against all‐trans‐retinal–induced toxicity and inhibits A2E formation
Source: J Cell Mol Med. 2016 Apr 12;20(9):1651–63. doi: 10.1111/jcmm.12857 (PMC4988284; doi:10.1111/jcmm.12857)

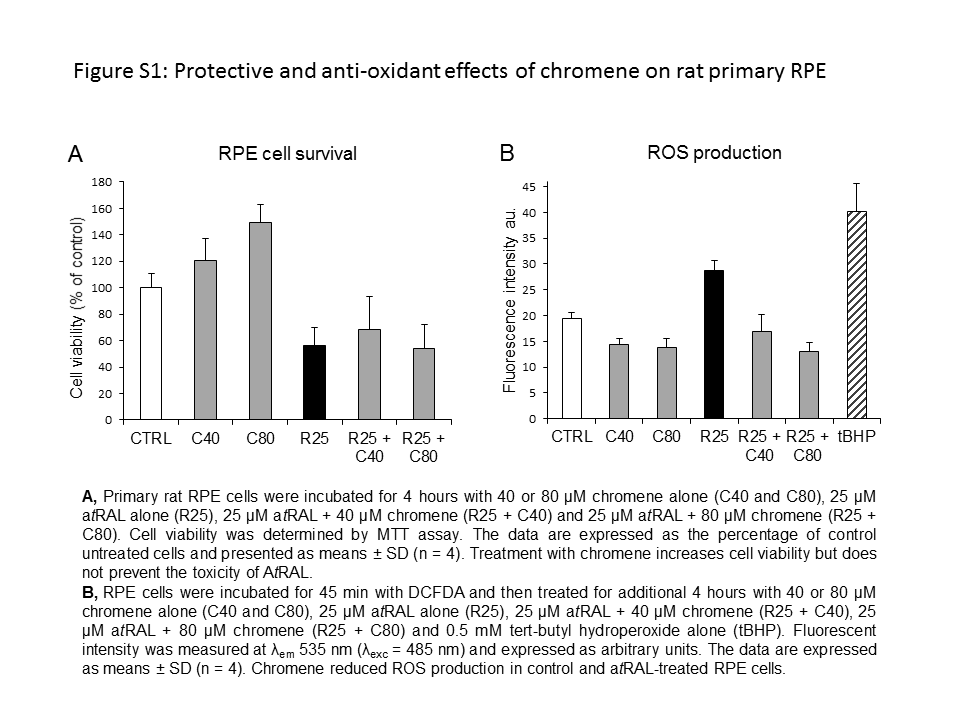

Supplement: Supplementary file 1 — Figure S1. Protective and anti‐oxidant effects of chromene on rat primary RPE. [file JCMM-20-1651-s001.tiff]
