# Supplementary material for: Phloroglucinol protects retinal pigment epithelium and photoreceptor against all‐trans‐retinal–induced toxicity and inhibits A2E formation
Source: J Cell Mol Med. 2016 Apr 12;20(9):1651–63. doi: 10.1111/jcmm.12857 (PMC4988284; doi:10.1111/jcmm.12857)
Supplement: Supplementary file 2 — Figure S2. Comparaison of A2E synthesis reaction and competition 1 reaction. [file JCMM-20-1651-s002.pdf]

Comparaison of A2E synthesis reaction and competition reaction C1.

UPLC-UV(PDA 200-800nm)-MS. – UPLC Accela qnd Masse spectrometer LCQFleet from Thermo scientific.
Column : Xbridge BEH C18 2.5uM, 2.1x100mm
Flow: 0.250ul/min
A: H2O, B: ACN
30/70 (0 to 20min)
30/70 to 0/100 (20 to 25 min)
0/100 (25 to 40min)
Dulition sample 20 time in H2O/CAN 30/70, filtration, Injection volume 20uL

A2E synthesis (1eq.ethanolamine , 2eq.atRAL)

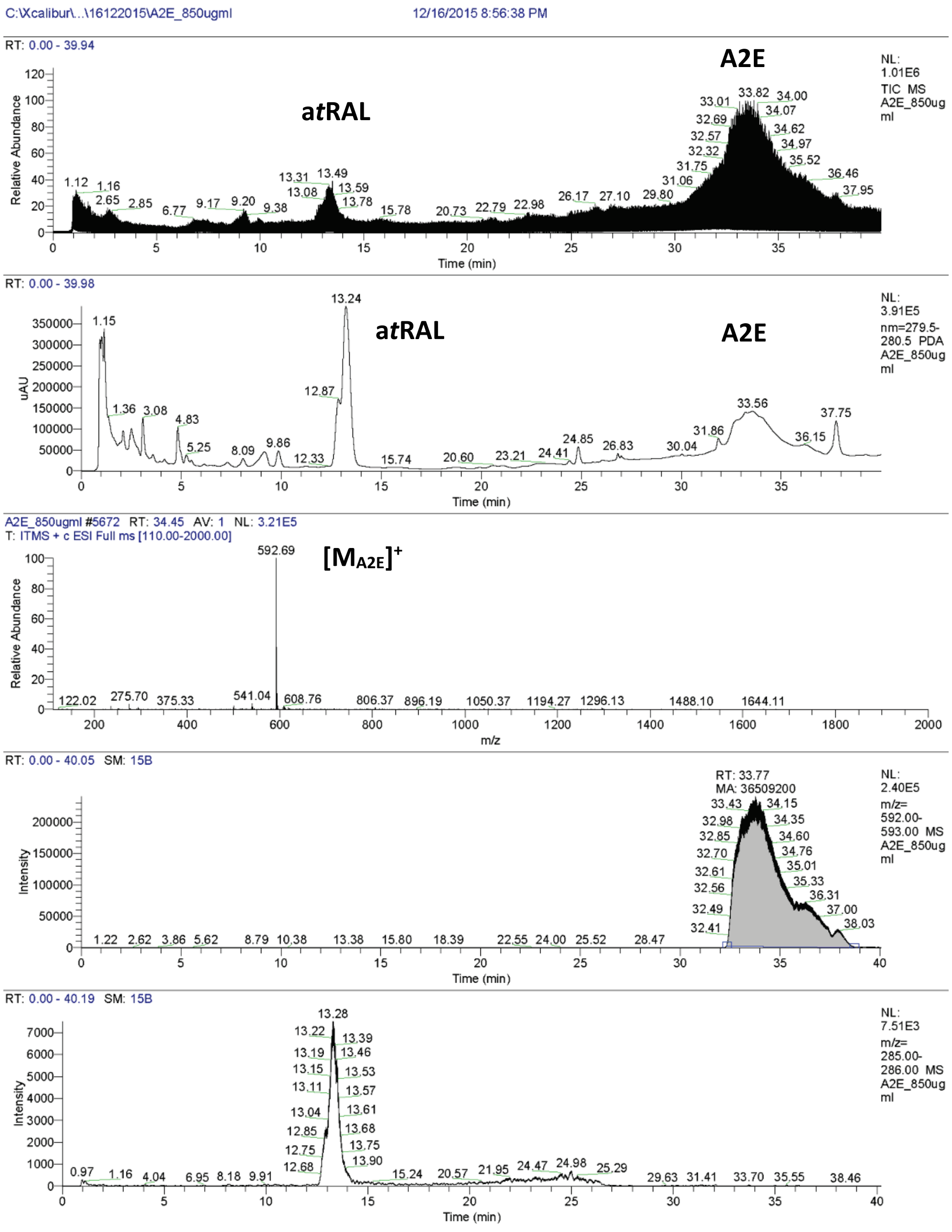

Total Ion Chromatogram (TIC)

UV (200-800m)

MS at rt: 34min

Filter : masse of A2E

Area under peak :36509200

Filter: masse of atRAL

Competition reaction C1 : (1eq.ethanolamine , 1eq. phloroglucinol, 2eq. atRAL)

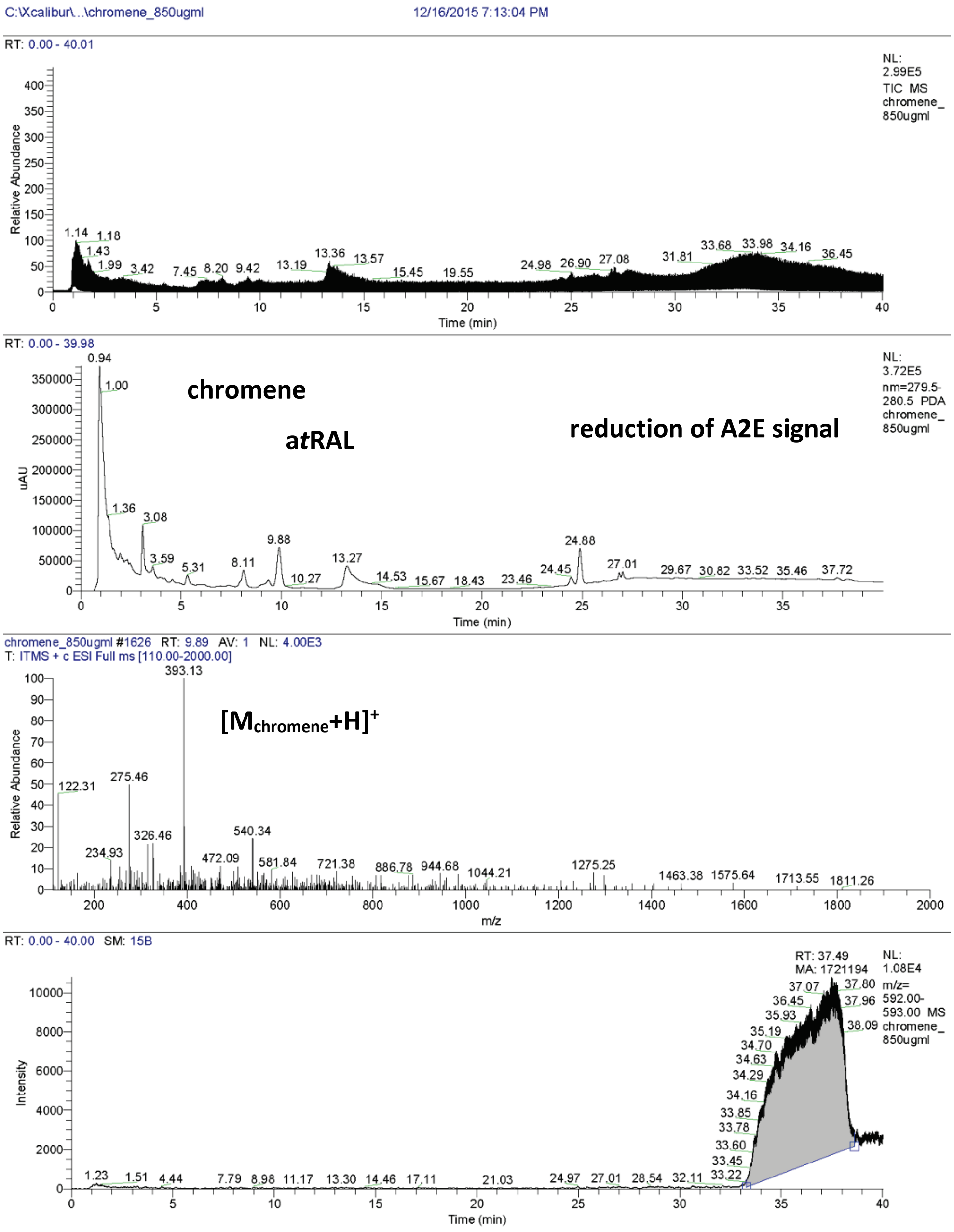

TIC

UV (200-800m)

MS at rt: 9.88 min

Filter: masse of A2E

Area under peak :1721194

Diminution of A2E and retinal in competition reaction.

Detection of the chromene in peak 9.88 min, m/z 393

No major additional pics are identified during UV or MS analysis.

Comparaison of A2E synthesis and competition reaction C1 by UV (300nm)

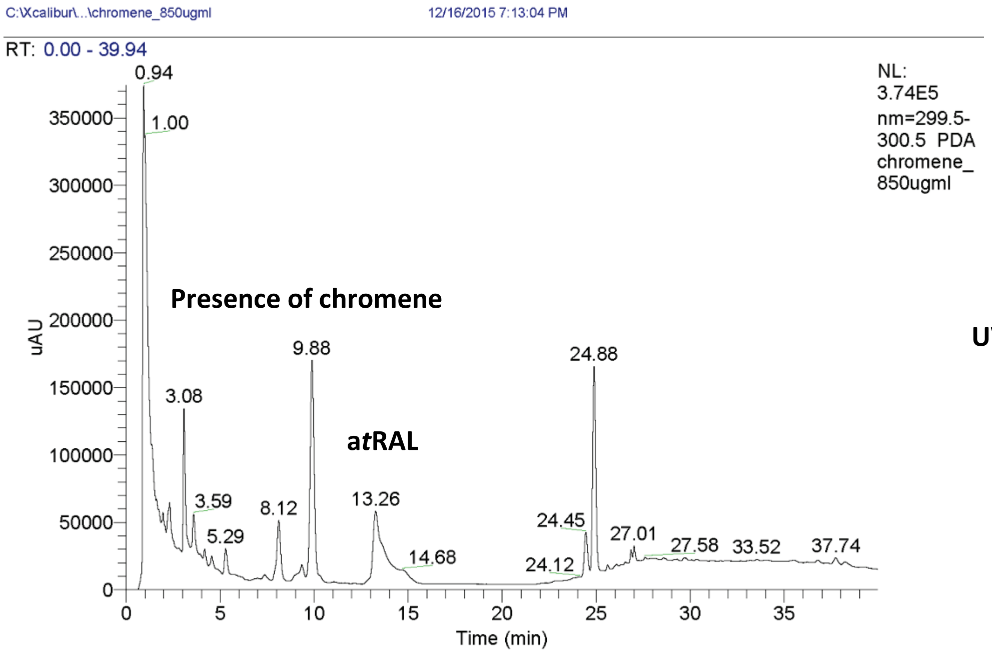

UV (300nm) C1

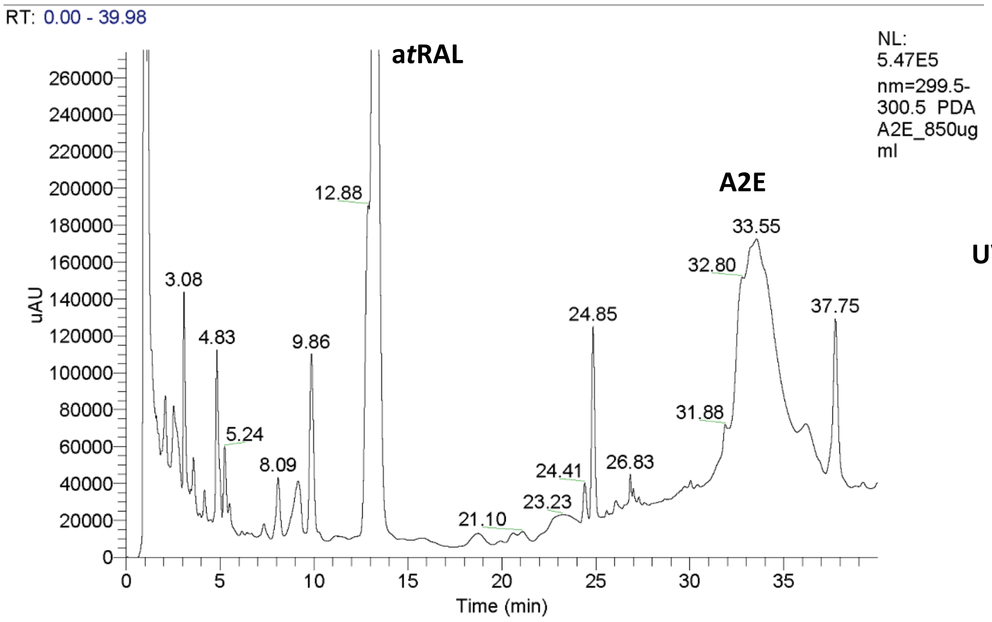

UV (300nm) A2E synthesis

Chromene UV signal at 9.8 min is not only the one of chromene compound since the peak at 9.86min was also present in analysis of the A2E synthesis reaction. However **$[M_{chromene}+H]^+$** ion signal is only visible in the competition reaction at 9.8 min .
